# Structural Dynamics of the Skin-Associated Microbiome of the Sea Cucumber *Holothuria scabra* During Integument Ulceration and Recovery

**DOI:** 10.1007/s00284-025-04475-9

**Published:** 2025-09-02

**Authors:** Yuanqiu He, Bovern Suchart Arromrak, Juan Diego Gaitan-Espitia

**Affiliations:** School of Biological Sciences, The Swire Institute of Marine Science, The University of Hong Kong, Hong Kong, China

## Abstract

Sea cucumbers are highly valued commercial species in the seafood industry. However, their hatchery and commercialization are constrained by the high mortalities experienced by these animals driven by environmental changes and physiological stress. Under such conditions, functional alterations in their immune system can induce integument ulcers that are later infected by opportunistic pathogens. Sea cucumbers, however, can display defense mechanisms that enable self-healing and recovery. It has been hypothesized that such mechanisms are assisted by changes in the skin-associated microbial community, enhancing the resilience of infected animal hosts. Here, we investigated the dynamics of the skin-associated microbiomes during infection and recovery of the tropical sea cucumber *Holothuria scabra*. Our findings reveal that the acclimation process for both, the host and the microbiome occurs simultaneously, with the latter gradually resembling the surrounding water environment after one month. The gradual healing of the sea cucumber skin and the structural changes in the associated microbiome were concurrent to the decline of pathogenic bacteria and the increase in the abundance of the family *Rhodobacteraceae*. The enrichment of some members from this group (genus *Ruegeria*) suggests a potential beneficial role in facilitating sea cucumber host recovery and acclimation. Our study provides valuable insights into the dynamics of the skin microbiome in sea cucumbers, highlighting the key role of this organismal trait for the resilience and overall health of the sea cucumber host.

**Supplementary Information:**

The online version contains supplementary material available at 10.1007/s00284-025-04475-9.

## Introduction

*Holothuria scabra*, commonly known as sandfish, is a tropical sea cucumber and a highly valued species in the seafood industry [1–3], just behind the temperate species *Apostichopus japonicus* [4]. The commercialization of *H. scabra* involves the transportation of live sea cucumbers across countries, from collection sites and hatcheries in South Asia to consuming places, primarily China and Singapore [5]. Such transportation is a major challenge due to the physiological stress and mortality induced to sea cucumbers [6, 7]. The environmental stressors (e.g., low oxygen, high temperature) faced by these animals during transportation can lead to skin degradation, evisceration, and ultimately death, factors with important socio-economic implications to the seafood industry [8, 9].

Previous studies have proposed diverse methods to improve the efficacy of live transportation of sea cucumbers, including seawater oxygen supersaturation [10], and cooling [8, 11]. Although these methods can reduce physiological stress during short-term transport, prolonged periods still result in detrimental effects such as oxidative stress [12], and the decline of immunity and survival [13]. Such negative effects have been linked to the skin ulceration syndrome (SUS), in which stressed sea cucumbers develop integument ulcers that are later infected by opportunistic pathogens that promote ulcer extension [14, 15]. Sea cucumbers, however, possess cellular defense mechanisms that enable self-healing after experiencing environmental stresses [12]. For instance, in *H. scabra*, skin ulceration can spontaneously disappear within 10 days when animals are under optimal environmental conditions [14]. This physiological resilience and skin recovery can be assisted by changes in the microbial community in which some taxa play beneficial roles for the sea cucumber host [16–19]. For example, some bacteria have been found to interact with the host during gut regeneration, showing inhibitory activities against pathogenic *Vibrio* in sea cucumbers [20, 21]. Similarly, bacteria such as *Bacillus licheniformis,* have shown probiotic properties reducing mortality, improving skin ulceration symptoms, and controlling residual pathogenicity [22]. Despite this progress, it remains unclear the structural and functional dynamics of the sea cucumber skin microbiome underpinning the recovery and resilience of infected animals. Moreover, it is unknown to what extent such dynamics are stochastic or deterministic processes modulated by the environment and/or the host.

The stability, resilience and recovery capacity of the skin microbiome are crucial to host homeostasis as these microbial communities contain both probiotic bacteria and opportunistic pathogens [23]. Therefore, investigating the dynamics of the skin-associated microbiomes during infection and recovery of sea cucumber hosts is an essential step for implementing strategies to mitigate the impacts of SUS and the negative effects of live transportation. Here, we tested this theoretical framework by assessing structural and functional changes during the infection and recovery post-live transportation in the sea cucumber *H. scabra*. Considering the homeostatic capacity documented for infected animals of this species, we hypothesize that temporal changes in the skin microbiome follow a deterministic process modulated by the physiological recovery of *H. scabra*. This research provides valuable insights regarding stress-response and acclimatory mechanisms of sea cucumbers during live transportation and commercialization in the seafood and aquaculture industry.

## Materials and Methods

### Animal Transportation and Aquarium Settings

A total of 18 *H. scabra* juveniles (body mass ~ 100 g; body length: 12 cm to18cm) were raised in a hatchery in Vietnam and transported from Vietnam to Hong Kong by flight. After arrival at the University of Hong Kong, animals were randomly allocated to two tanks, each one filled with commercial aquaculture sand and 40 L natural seawater, connected to a filtration system (Dophin CF800). The body condition of each animal was visually assessed at the arrival, and photographically recorded in order to detect wounds, white skin ulceration or signals of gut evisceration. Temperature was maintained at 29 °C using aquarium heaters. Daily maintenance included the removal the feces, refilling 10 L of seawater and animal feeding with a commercial diet (Dalian Scitech Aquafeed Co. Ltd). Animals were continuously monitored for one month.

### Skin Sample Collection

Sterilized cotton swabs were used to collect skin samples from the dorsal segment of the sea cucumbers. After sampling, swabs were placed in 1.5 mL sterilized tubes and stored at -80ºC until DNA extraction. Photos of sea cucumbers were taken after the sample collection to record animal body condition. The individuals with gut evisceration were recognized by flat and shrunken body with a sunken line appeared in the middle dorsal skins at each time point. One-liter water samples were collected from each aquarium and then filtered (0.22 μm) for the assessment of environmental microbial communities. Filters were then stored at – 80 °C for subsequent use. Skin samples were collected on day 1, 4, 7, 15, 23, and 30 before the daily maintenance of the aquarium. Water samples were collected as the reference on day 7, 15, and 30.

### DNA Extraction, Amplification and Sequencing

Swaps and filters were processed for DNA extraction using Qiagen DNeasy Blood and Tissue Kits according to the manufacturer’s instructions. DNA samples were sent to Novogene (HK) Co., Ltd for 16S rRNA gene amplification sequencing. The quality of DNA and the amplified products were checked with 2% agarose gel. 16S rRNA gene amplicon sequencing was conducted in Illumina Novaseq 6000 with primer 338F (5′-ACTCCTACGGGAGGCAGCA-3′) and 806R (5′-GGACTACHVGGGTWTCTAAT-3′) using PE 250 strategy. Raw reads were deposited in the NCBI BioProject with accession codes PRJNA1017642.

### Bioinformatic and Statistical Analyses

Raw reads filtration and barcode removal were done by Trimmomatic v0.33. Qiime2 (version 2022.2) was used for removing primer sequences (cutadpt), denoise and assembled paired-end sequences (DADA2). The obtained amplicon sequence variants (ASVs) were assigned to the taxon with the Naive Bayes classifier using Silva reference sequences (classifier_silva_13_8_99). The microbial feature table generated from Qiime 2 was imported to R (version 4.3.1) for further analysis. The package vegan was used in alpha diversity and beta diversity (bray method) analysis with the flattening feature table. The linear model was conducted in R with the alpha diversity index of the two variables (time and types) as fixed factors. As the sea cucumbers from the two tanks were randomly selected for sampling at each time point, each skin sample got a unique animal ID, which were deemed as random factor in the analysis. The Principal Coordinate Analysis (PCoA) was plotted for beta diversity and the PERMANOVA multiple comparisons were used to measure the significant difference among groups with 999 permutations based on Bray Curtis distance. Permutation test for homogeneity of multivariate dispersions for factors were calculated by package ‘vegan’ with 999 permutations based on Bray Curtis distance. The alluvial plot was generated by package ‘ggalluvial’ and the families whose relative abundance above 2% in any sampling dates were selected for the plot and the average relative abundance of the replicates were used). Differential abundance analysis was performed in R by package ANCOMBC at family level (the ASVs that existed in only one sample or its total counts less than 10 in all samples were filtered out). ANCOM-BC analysis was conducted twice with different control groups. In order to find out the families that had changed in skin microbiome after the one-month acclimation, we compared the skin samples on day 1 (set as reference group) and day 30. The families would be selected to plot figure by package ggplot2 if they showed significant difference (return ‘True’ in ANCOM-BC result) and their absolute value of log-fold-change above three. To figure out the different taxa between the skin microbiome and the water microbiome, ANCOM-BC analysis was conducted again between the skin samples and water samples on day 30, and the skin samples on day 30 were selected as reference. The package ggplot2 and dplyr were used to show the result from ANCOM-BC and the change of relative abundance of pathogenic bacterial taxa with time. Raw reads were deposited in the NCBI BioProject with accession codes PRJNA1017642.

## Results

### Initial Assessment of Body Condition

At the arrival at Hong Kong, animals showed signs of stress evidenced by gut evisceration and small skin ulcers on the dorsal part (Fig. S1A). These animals were randomly separated in the two experimental tanks. The body condition at each timepoint was recorded by photos in Fig. S1B and the individuals with special body conditions were marked by symbols (Fig. S1B). The body shape of individuals who had gut evisceration differed from the others during the first two weeks. However, the individual difference gradually reduced after day 23 because of the gut regeneration.

### Acclimation of Sea Cucumber Skin Microbiome

**Fig. 1:**
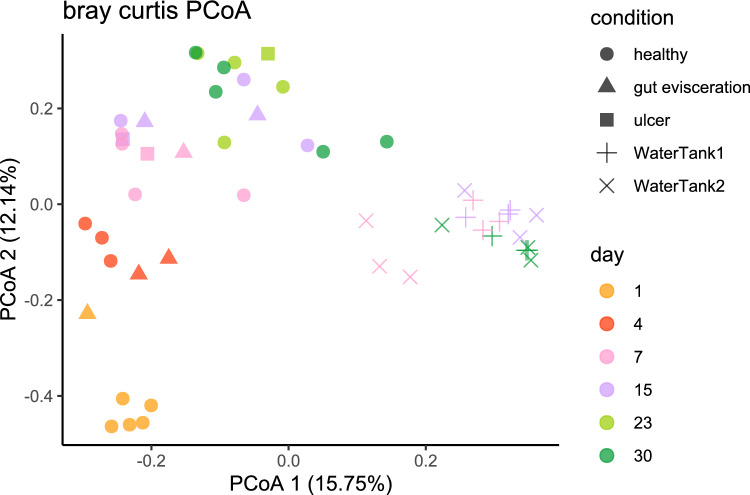
The principal coordinate analysis (PCoA) plot of skin microbiome with time. (Individuals were marked as different the condition)

All animals survived the one-month acclimation period after their arrival. In the first week, sea cucumbers were inactive, gathered in groups under the sand. However, most individuals became active from day 7, crawling and feeding over the sediment. Such behavior was consistent until the end of the acclimation. The 16S rRNA gene amplicon sequencing generated 11,473,924 raw reads (quality score > 20), which were assigned to 20,067 ASVs (Table S3). Classes such as *Alphaproteobacteria* (37.5 ± 12.4%), *Gammaproteobacteria* (26.4 ± 12.2%), *Bacteroidia* (17.0 ± 8.2%), were dominant taxa in the sea cucumber skin microbiome. A temporal trend was observed in the principal coordinate analysis (PCoA) plot (Fig. 1), in which the skin microbiome was gradually acclimated to the local conditions in Hong Kong. On day 1, 4, 7, 15, the skin microbiome showed major, but consistent, transitions that were less accentuated with time (*P*_day1 vs. day4_ < 0.05, *P*_day4 vs. day7_ < 0.05 and *P*_day7 vs. day15_ < 0.05 in Pairwise comparisons of PERMANOVA; Table S1). From day 15, the skin microbiome started to resemble more the water microbial communities with less marked differences (*P*_day15 vs. day23_ > 0.05, *P*_day23 vs. day30_ > 0.05) among sampling points in this second temporal phase. However, the microbial structure from individuals that exhibited gut evisceration or skin ulcers, showed slightly departures from the microbial community profiles documented in healthy individuals (Fig. 1).

### Dynamic Bacterial Community Diversity

**Fig. 2:**
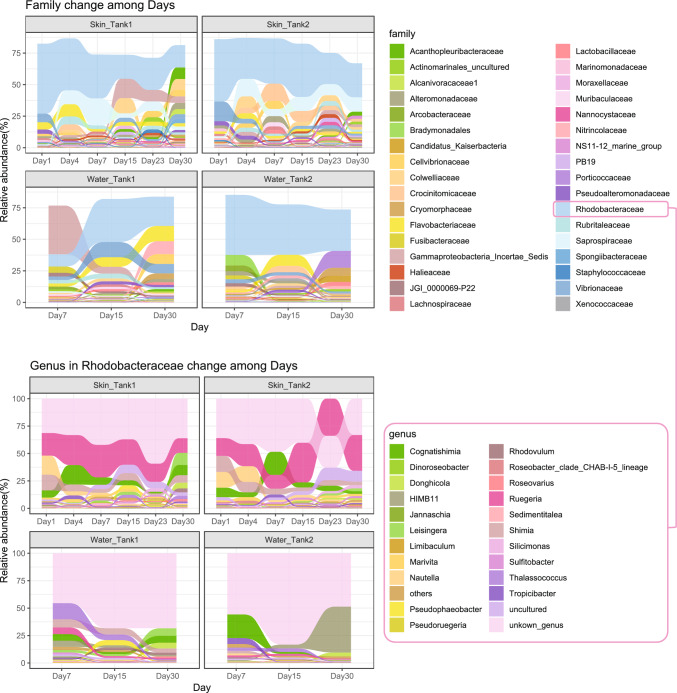
Alluvial plot showed the change of families and genus among days. (Families with relative abundance greater than 2% are shown in the upper part of the plot. The change of genera belong to the family *Rhodobacteraceae* is shown in the bottom part of the plot)

The Shannon and ACE indexes of the skin microbiome showed significant differences compared to the water microbiome (*P* < 0.05) (Figs. S2 and S3). However, no significant changes in alpha diversity indexes (Shannon, ACE) of the skin microbiome were detected during the one-month acclimation. Despite the lack of differences in alpha diversity through time, we found temporal changes in beta diversity in the skin microbiome during the acclimation (Fig. 1). Non-significant *P* value was obtained from Permutation test for homogeneity of multivariate dispersions for each factor (*P*_date_ = 0.208 and *P*_sample type_ = 0.246). In addition, marked temporal changes were observed in the major bacteria taxa (i.e., families with relative abundance > 2%) (Fig. 2). Family *Rhodobacteraceae* was the dominant group in both the skin (average relative abundance is 33.84 ± 11.42) and water (average relative abundance is 31.21 ± 12.14) microbiomes (Fig. 2). This family was mainly represented by an unidentified genus and the *Ruegeria* genus (Fig. 2). On day 1, the general profile of the two tanks was similar, dominated by *Ruegeria*, *Nautella*, *Shimia* and *Cognatishimia*. After one-month acclimation, more diverse genera were found in the skin microbiome, while *Ruegeria* remained as the most dominant group. However, the relative abundance of the genus *Ruegeria* in the water samples was significantly lower than the skin samples (ANCOM-BC analysis; *P* < 0.05), indicating that this genus was specifically enriched in sea cucumber skin microbiome and kept dominant during the acclimation.

### Differential Analysis of Microbiome After Acclimation

**Fig. 3:**
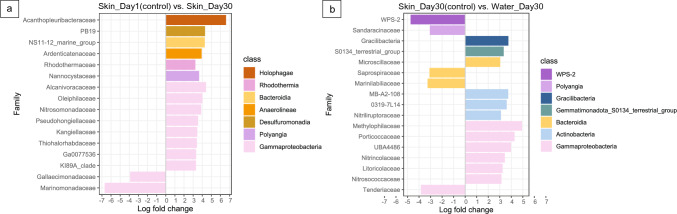
The log-fold-change from the comparison of skin microbiome and water microbiome. (**A**: the comparison between the skin microbiome on day 1 and day 30. The family with positive log-fold-change indicated it enriched on day 30; **B**: the comparison between the skin microbiome and water microbiome on day 30, the family with minus log-fold-change indicated it enriched in skin; Detailed result was shown in Table S2)

From the result of ANCOM-BC (Table S2), we found that 106 families in the skin microbiome significantly changed on day 30, compared to the first day. Among them, 79 families were significantly enriched in the skin microbiome, while 27 families significantly decreased (*P* < 0.05). After one-month acclimation, the family *Acanthopleuribacteraceae* was enriched in the skin microbiome with the highest log-fold-change. Another five families from various classed and eight families from class *Gammaproteobacteria*, also increased their abundance in skin microbiome through time. Two families, *Gallaecimonadaceae* and *Marinomonadaceae* had sharp decrease with the time. The ANCOM-BC analysis found 99 distinct bacteria families in the comparison between the skin microbiome and water microbiome on day 30 (skin samples on day 30 was the reference group). 26 families were significantly enriched in the sea cucumber skin microbiome, and five families, including WPS-2, *Tenderiaceae*, *Marinilabiliaceae*, *Saprospiraceae* and *Sandaracinaceae* had high log-fold-change (above 3) among them (Fig. 3B).

### Change of Pathogenic Bacterial Taxa with Time

**Fig. 4:**
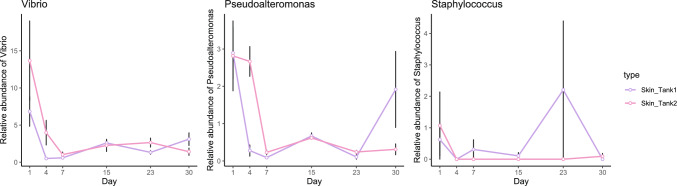
The relative abundance of pathogenic bacterial taxa from the skin microbiome during the recovery

The relative abundance of two pathogenic bacteria from the skin microbiome was studied during the recovery (Fig. 4). In our study, the genera *Shewanella* only appeared in few water samples with low relative abundance. The genus Vibrio had the highest relative abundance on day 1 (averaged 6.83 ± 2.84% in tank1 and 13.65 ± 7.60% in tank2) and decreased during the first week of the acclimation. This group kept a stable proportion (~ 1–3%) in both tanks, and finally showed the averaged relative abundance of 3.11 ± 0.85% in tank1 and 1.42 ± 0.69% in tank2. The genus *Psedoalteromonas* had the similar change as Vibrio, dropping from averaged 2.85 ± 1.16% on day 1 to 0.19 ± 0.09% on day 23. On day 30, the relative abundance of genus *Psedoalteromonas* increased back to 1.92 ± 1.02% in tank1 while the no significant changes in tank2. The genus Staphylococcus showed low abundance on day 1 (averaged relative abundance 0.84 ± 1.21%), with an stochastic increase (2.20 ± 2.19%) on day 23, to finally decline (0.06 ± 0.11%) on day 30 (Fig. 4). The relative abundance of these pathogenic genera in the skin microbiome was high in the first week (stress post-transport), but dropped with time, indicating a potential restriction in the growth of pathogenic bacteria during the host acclimation and recovery.

## Discussion

The high sensitivity of sea cucumbers to environmental changes represents a major challenge for the live transportation and commercialization of these animals [6]. One of potential strategy to enhance sea cucumber’s resilience and survival in these processes, is by aiding in their acclimation and recovery post-transportation. In this study, we investigated the temporal dynamics during the acclimation process of both, the sea cucumber host and its associated skin microbiome, after the physiological stress induced by live transportation and handling. Our findings reveal a deterministic acclimation process in which the phenotypic recovery of the sea cucumber host occurs simultaneously to changes in the skin microbiome. Moreover, we observed that sea cucumbers typically complete their acclimation and recovery from transportation stress within a few weeks (Fig. S1). In parallel, the skin microbiome samples gradually resemble the water sample over time, with the acclimation rate slowing down after 15 days, indicating the conclusion of the skin microbiome acclimation process (Fig. 1). Additionally, there were no significant changes in the alpha diversity index of the skin microbiome, suggesting that acclimation does not involve an increase or decrease in taxon diversity, but rather a replacement by the ambient water microbiome. The simultaneous acclimation of the skin microbiome and the host highlights the potential synchrony in their responses to the new environment. Similar patterns have been observed in other marine organisms. For instance, in corals, temporal changes in associated microbiomes follow their host acclimation responses to cope with thermal stress [24]. Likewise, bacterial communities in fish also change synchronously to their host during the acclimation process to salinity alterations [19]. Thus, the co-acclimation process can be beneficial for both the host and its microbiome when confronted with a new environment.

During the acclimation period, we observed that gradual changes in the skin microbiome that gradually resembled the surrounding seawater microbial community. However, it is important to note that the family *Rhodobacteraceae* remained the most abundant taxon throughout the acclimation process. This family was primarily represented by an unidentified genus and the *Ruegeria*. Interestingly, the abundance of the *Ruegeria* genus in the bacteria community of the ambient seawater was lower compared to that on the sea cucumber skin (Fig. 2). This suggests that the sea cucumber enriches the abundance of *Ruegeria*, which may potentially contribute to the host in various ways. The family *Rhodobacteraceae* has also been identified as a core microbial group in other marine organisms such as the sea star *Odontaster validus* [25] and coral [26]. However, the specific mechanisms by which the family *Rhodobacteraceae* supports the sea cucumber as core microbes in the skin during acclimation are still unclear and require further investigation.

By the end of the acclimation period (i.e., day 30), we observed an increase in the abundance of several families in the skin microbiome of the sea cucumbers. For instance, the family *Acanthopleuribacteraceae* showed enrichment during the last two weeks of the acclimation period (Table S2A). This family is a common animal-associated marine bacteria known for its ability to produce various natural products [27]. On the other hand, two families, *Gallaecimonadaceae* and *Marinomonadaceae*, belonging to the class *Gammaproteobacteria*, exhibited a sharp decrease over time (Table S2B). Interestingly, these two families were not found in the ambient water microbiome, suggesting that they were replaced by the water microbiome during the acclimation process. The skin microbiome eventually established stable bacterial communities, and the replacement by the ambient microbiome was completed by the end of the acclimation period. Although the bacterial communities from the skin and water became more similar over time, they still had distinct enriched bacterial groups (Fig. 3B). The skin microbiome exhibited higher diversity compared to the ambient water microbiome (Fig. S3). Such pattern has also been reported in reef fish, where the skin microbiome hosts twice as many classes and phyla compared to the seawater [28]. Therefore, the relationship between the skin microbiome and the seawater microbiome is correlative, and hosts have the ability to selectively enrich or eliminate specific bacterial groups through cellular and metabolic processes [29].

Bacteria such as *Vibrio*, *Pseudoalteromonas*, *Shewanella*, and *Staphylococcus* have been identified as pathogens in diseased sea cucumbers [30–32]. In our study, we observed that the relative abundance of these pathogenic genera in the skin microbiome was initially high in the first week but decreased over time (Fig. 4). This suggests that opportunistic pathogenic bacteria may infect vulnerable hosts during periods of environmental stress. However, as the host gradually recovers during the acclimation process, the infection by these pathogenic bacteria is inhibited. Therefore, it is unlikely that pathogenic bacteria are the primary cause of host mortality. A study investigating sea cucumber skin ulceration disease (SKUD) also found that the development of SKUD is not solely attributed to the high proportion of pathogens, but rather may be influenced by environmental factors such as the host’s exposure to low temperatures [14]. Therefore, the key factor for successful acclimation lies in the resilience and overall health of the host, rather than the presence of opportunistic pathogens.

In conclusion, our study provides valuable insights into the dynamics of the skin microbiome in sea cucumbers following live transportation. We observed that the acclimation process to the new environment occurs not only in the host but also in its skin microbiome, taking approximately three weeks after live transportation. Therefore, it is crucial to provide an optimal environment for sea cucumbers to support their recovery from transportation stress. Interestingly, specific bacterial taxa, such as the genus *Ruegeria*, were found to be enriched in the sea cucumber skin microbiome, suggesting their potential role in facilitating host acclimation. However, the underlying mechanisms remain unclear and warrant further investigation.

## Supplementary Information

Below is the link to the electronic supplementary material.

Supplementary file1 (PDF 1348 KB)

Supplementary file2 (PDF 239 KB)

Supplementary file3 (PDF 37 KB)

Supplementary file4 (CSV 3 KB)

Supplementary file5 (XLSX 605 KB)

Supplementary file6 (XLSX 13 KB)

## Data Availability

Raw reads were deposited in the NCBI BioProject with accession codes PRJNA1017642. Scripts and data have been deposited in the HKU FigShare Repository https://figshare.com/s/f846cc7b9adfc44997f9. 10.25442/hku.29222210.
