# Supplementary material for: Structural Dynamics of the Skin-Associated Microbiome of the Sea Cucumber Holothuria scabra During Integument Ulceration and Recovery
Source: Curr Microbiol. 2025 Sep 2;82(10):489. doi: 10.1007/s00284-025-04475-9 (PMC12405312; doi:10.1007/s00284-025-04475-9)
Supplement: Supplementary file 1 — Supplementary file1 (PDF 1348 KB) [file 284_2025_4475_MOESM1_ESM.pdf]

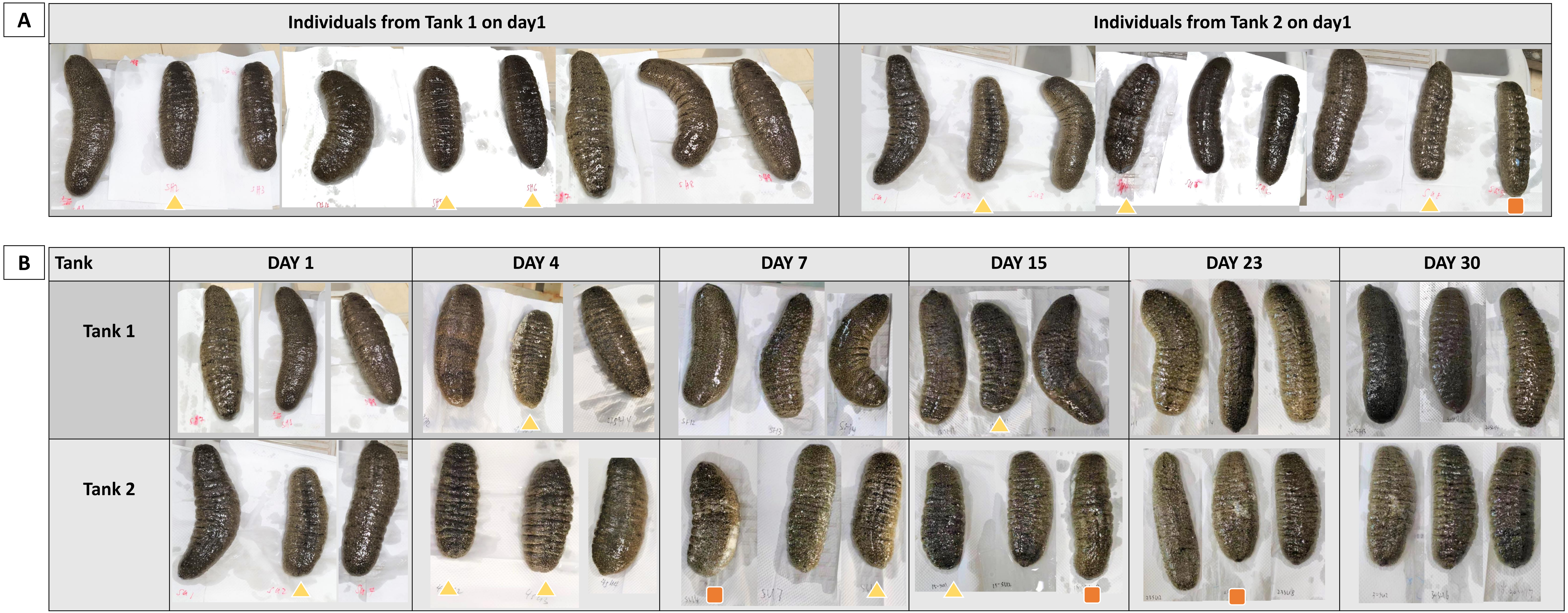

Fig.S1. Photos of sea cucumber

(A: Photos of sampled sea cucumber on day 1. B: Photos of sampled sea cucumber at each time point. Individuals with gut evisceration and ulcer are marked with "▲" and "■", respectively.)
