# Supplementary material for: Structural Dynamics of the Skin-Associated Microbiome of the Sea Cucumber Holothuria scabra During Integument Ulceration and Recovery
Source: Curr Microbiol. 2025 Sep 2;82(10):489. doi: 10.1007/s00284-025-04475-9 (PMC12405312; doi:10.1007/s00284-025-04475-9)
Supplement: Supplementary file 2 — Supplementary file2 (PDF 239 KB) [file 284_2025_4475_MOESM2_ESM.pdf]

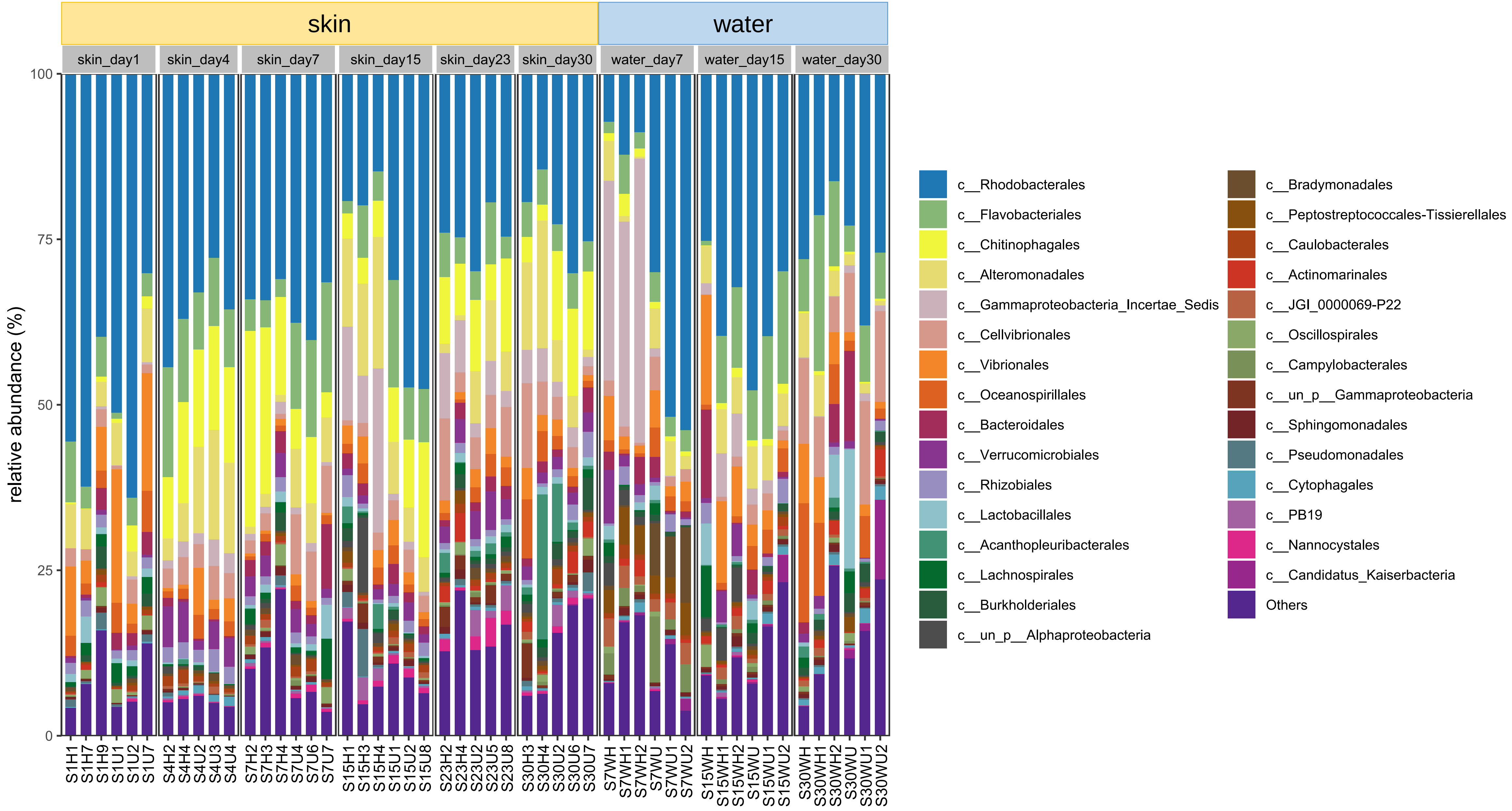

Fig. S2 The microbial profile of the skin microbiome at order level
(Water samples were showed as reference)
