# Supplementary figures and images for: Structural Dynamics of the Skin-Associated Microbiome of the Sea Cucumber Holothuria scabra During Integument Ulceration and Recovery
Source: Curr Microbiol. 2025 Sep 2;82(10):489. doi: 10.1007/s00284-025-04475-9 (PMC12405312; doi:10.1007/s00284-025-04475-9)

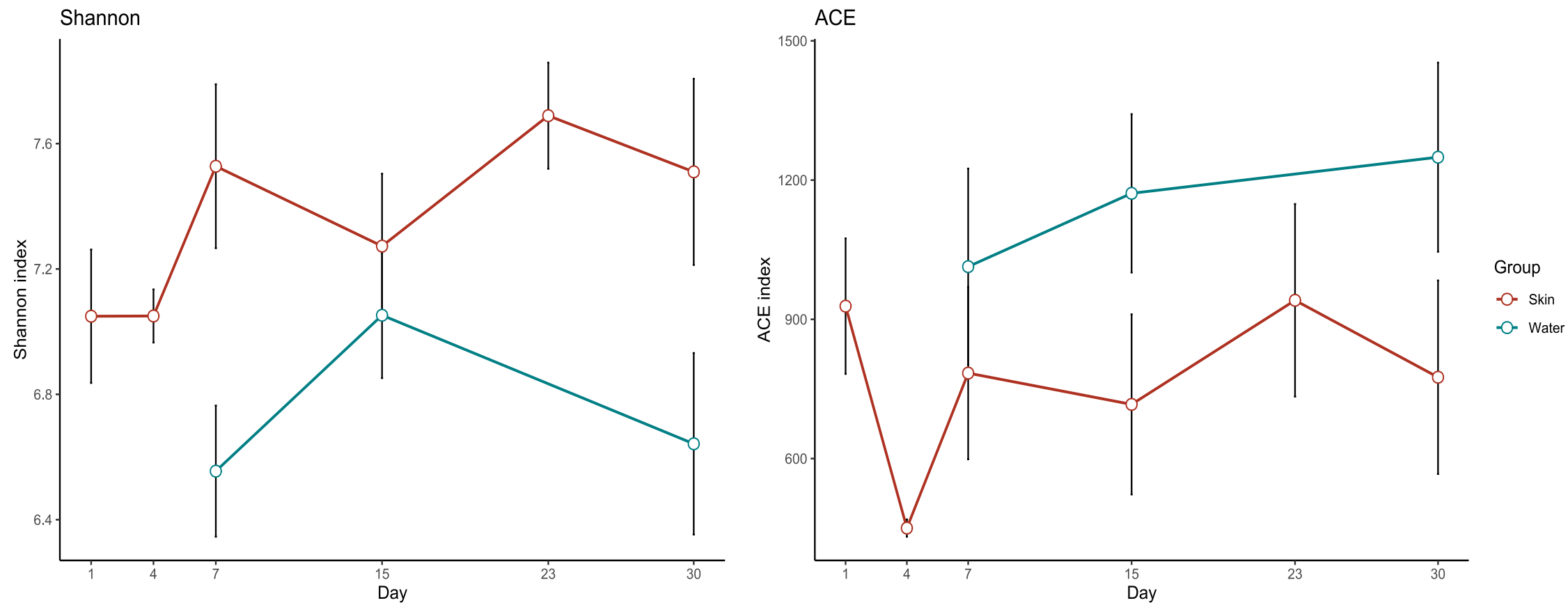

Fig. S3 The change of alpha diversity in skin microbiome during one-month acclimation

Supplement: Supplementary file 3 — Supplementary file3 (PDF 37 KB) [file 284_2025_4475_MOESM3_ESM.pdf]
